# Differential effects of minocycline on microvascular complications in murine models of type 1 and type 2 diabetes

**DOI:** 10.15761/jts.1000431

**Published:** 2020-06-16

**Authors:** Stephanie A. Eid, Phillipe D. O’Brien, Lucy M. Hinder, John M. Hayes, Faye E. Mendelson, Hongyu Zhang, Samanthi Narayanan, Steven F. Abcouwer, Frank C. Brosius, Subramaniam Pennathur, Masha G. Savelieff, Eva L. Feldman

**Affiliations:** 1 Department of Neurology, University of Michigan, Ann Arbor, MI, 48109, USA; 2 Division of Nephrology, Department of Internal Medicine, University of Michigan, Ann Arbor, MI, 48109, U.S.A.; 3 Department of Ophthalmology and Visual Sciences, University of Michigan, Ann Arbor, MI, 48105, U.S.A.; 4 Departments of Molecular and Integrative Physiology, University of Michigan, Ann Arbor, MI, 48109, U.S.A.

**Keywords:** diabetes, minocycline, nephropathy, peripheral neuropathy, retinopathy

## Abstract

Diabetes is a global healthcare problem associated with enormous healthcare and personal costs. Despite glucose lowering agents that control glycaemia, both type 1 (T1D) and type (T2D) diabetes patients often develop microvascular complications that increase morbidity and mortality. Current interventions rely on careful glycemic control and healthy lifestyle choices, but these are ineffective at reversing or completely preventing the major microvascular complications, diabetic peripheral neuropathy (DPN), diabetic retinopathy (DR), and diabetic kidney disease (DKD). Minocycline, a tetracycline antibiotic with anti-inflammatory and anti-apoptotic properties, has been proposed as a protective agent in diabetes. However, there are no reported studies evaluating the therapeutic efficacy of minocycline in T1D and T2D models for all microvascular complications (DPN, DR, and DKD). Therefore, we performed metabolic profiling in streptozotocin-induced T1D and db/db T2D models and compared the efficacy of minocycline in preventing complications to that of insulin and pioglitazone in both models. Minocycline partially ameliorated DR and DKD in T1D and T2D animals, but was less effective than insulin or pioglitazone, and failed to improve DPN in either model. These results suggest that minocycline is unlikely to improve outcomes beyond that achieved with current available therapies in patients with T1D or T2D associated microvascular complications.

## Introduction

Both type 1 diabetes (T1D) and type 2 diabetes (T2D) are associated with complications including diabetic peripheral neuropathy (DPN), diabetic retinopathy (DR), and diabetic kidney disease (DKD) [1]. Glycemic control is only partially effective for preventing complications [2]. Thus, a better understanding of disease pathophysiology is crucial for developing effective therapies.

Using T1D and T2D mouse models, we and others have explored transcriptomic changes in complication-prone tissues and identified pathogenic pathways, including inflammation [3], oxidative stress [4], apoptosis [5], and mitochondrial dysfunction [6]. These observations defined potential therapeutic targets that may alleviate end-organ damage.

Minocycline, a tetracycline antibiotic, exerts a wide range of biological effects independent of its antimicrobial properties that make it an attractive therapeutic agent for diabetic complications. It has anti-inflammatory, anti-apoptotic, and anti-angiogenic actions, as well as neuroprotective effects [7]. It has been reported to improve DPN [8] and neuropathic pain [9], DR [10,11], and DKD [12,13], possibly due to anti-inflammatory, anti-oxidant, and anti-apoptotic actions [10–13]. Yet, there are no reported studies that explore the simultaneous effects of minocycline on all three complications in both T1D and T2D mouse models.

**Figure 1.**
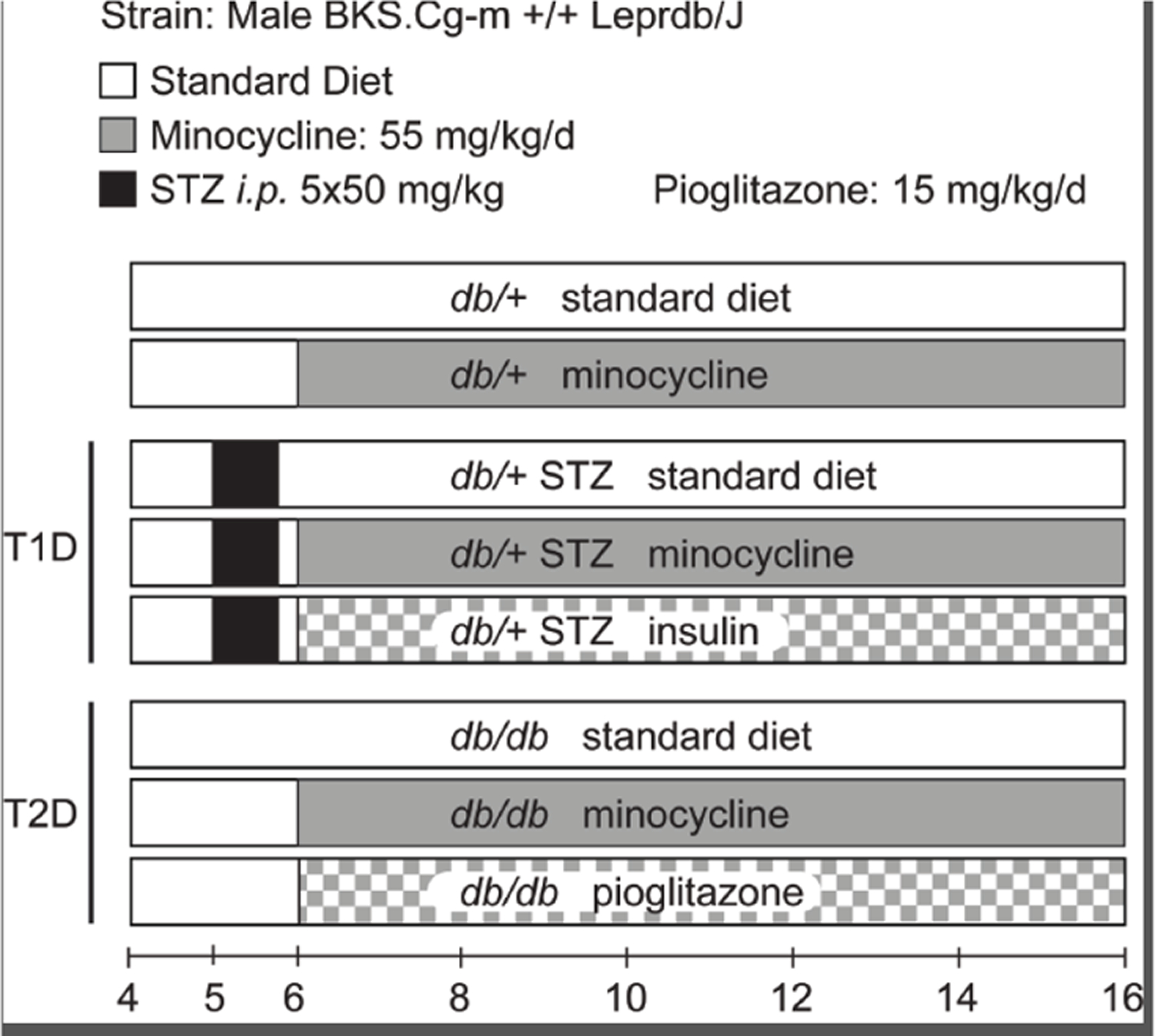
Study design. The study consisted of eight groups: (i) untreated *db/+* controls, (ii) minocycline-treated *db*/+ controls, (iii) untreated T1D *db*/+ STZ mice, (iv) minocycline-treated T1D *db*/+ STZ mice, (v) insulin-treated T1D *db*/+ STZ mice, (vi) untreated T2D *db*/*db* mice, (vii) minocycline-treated T2D *db*/*db* mice, and (viii) pioglitazone-treated T2D *db*/*db* mice. Untreated cohorts just ate standard chow. Minocycline dose was 55 mg/kg/day, and pioglitazone dose was 15 mg/kg/day. Treatment started in mice aged 6 weeks and lasted 10 weeks till study conclusion when mice were aged 16 weeks. Body weight and fasting blood glucose (FBG) levels were recorded every two weeks. At 16 weeks, metabolic, DPN, DR, and DKD phenotyping was performed

Herein, we evaluated minocycline treatment efficacy on DPN, DR, and DKD in well-established T1D and T2D models in comparison to insulin or pioglitazone, commonly prescribed to diabetic patients (Figure 1). We found that minocycline did not improve DPN, although partially it ameliorated DR and DKD in T1D and T2D animals, respectively.

## Materials and methods

### Animals

Male C57BLKS (BKS) db/+ mice (control) and BKS db/db mice were purchased from Jackson Laboratories (BKS.Cg-Dock7m +/+ Leprdb/J, Stock No: 000642; Bar Harbor, ME) at 4 weeks of age. Animals were maintained in specific-pathogen-free housing provided by the University of Michigan Unit for Laboratory Animal Medicine and given access to water and chow ad libitum. All protocols were carried out in accordance with the guidelines outlined by the Diabetes Complications Consortium (http://www.diacomp.org) and the National Institutes of Health’s (NIH) Guide for the Care and Use of Laboratory Animals (8th Edition). All protocols were approved by the University of Michigan’s Institutional Animal Care and Use Committee.

Animal models and study design: To induce T1D, 5-week-old db/+ mice were injected intraperitoneally with 50 mg/kg streptozotocin (STZ, Sigma Aldrich, St Louis, MO) dissolved in citrate buffer (pH 4.5), for 5 consecutive days (db/+ STZ) [14]. Leptin signaling deficient db/db mice were used as a T2D model [14]. Diabetes was defined as fasting blood glucose (FBG) levels over 300 mg/dL [15]. Starting at 6 weeks of age, mice were randomly assigned to minocycline (db/+ MINO, db/+ STZ MINO, db/db MINO) or pioglitazone (db/db PIO) (Figure 1) treatment groups and fed AIN-76A standard chow supplemented with 55 mg/kg/day minocycline or 15 mg/kg/day pioglitazone, respectively. Minocycline and pioglitazone were both purchased from Michigan Medicine, University of Michigan, and compounded into AIN-76A by Research Diets (New Brunswick, NJ). All other groups were maintained on a standard chow diet (untreated). Insulin was administered via LinBit (both from LinShin, Toronto, Canada) according to the manufacturer’s instructions to the db/+ STZ insulin group starting at 6 weeks of age, with implants replaced every 4 weeks until study termination (i.e., at 10 and 14 weeks). The study duration was 10 weeks and ended when the animals were aged 16 weeks. After phenotyping for each complication, mice were euthanized with 150 mg/kg sodium pentobarbital administered intraperitoneally. Blood was collected from the superior vena cava for glycated hemoglobin (%HbA1c) analysis and plasma processing. Hind paws were isolated for intraepidermal nerve fiber density (IENFD) analysis.

Metabolic phenotyping: Animals were weighed every 2 weeks. Fasting blood glucose (FBG) was measured using an AlphaTRAK glucometer (Zoetis, Parsippany-Troy Hills, NJ) every 2 weeks throughout the experimental period. %HbA1c was measured by ELISA at study termination using the manufacturer’s instructions (Mouse Hemoglobin A1c Assay Kit, cat# 80310; CrystalChem, Elk Grove Village, IL). Serum lipid profiles were analyzed by the Mouse Metabolic Phenotyping Center (MMPC) at Cincinnati (University of Cincinnati Medical Center, OH, USA; www.mmpc.org).

DPN phenotyping: DPN was measured using the protocols outlined by the Diabetes Complications Consortium (www.diacomp.org/shared/protocols.aspx). Nerve conduction velocities (NCVs) were assessed as a measure of large-fiber nerve function in sural sensory and sciatic-tibial motor nerves at the termination of the study as previously described [16]. IENFD is an anatomical measure of small nerve fibers. Fixed hind paw plantar tissue was stained with a pan-axonal marker, ubiquitin C-terminal hydrolase L1 (UCHL1; 1:2000; Proteintech, Rosemont, IL) [17]. Three random fields per mouse were imaged (1024×1024 pixel resolution, 3.3 µm optical section thickness, FluoView 500 confocal microscope, 20×1.2 objective, Olympus, Tokyo, Japan). MetaMorph (Molecular Devices, San Jose, CA) flattened ten images per stack, and IENFD was represented as fiber counts per mm.

DR phenotyping: DR was evaluated using protocols from the Diabetes Complications Consortium. Briefly, retinal DNA fragmentation was measured at the study termination by apoptotic DNA cleavage ELISA (Cell Death Detection, Roche Applied Science, Indianapolis, IN) and normalized to retinal wet weight [18].

DKD phenotyping: DKD was assessed using protocols from the Diabetes Complications Consortium and as previously reported [19]. Urinary albumin-to-creatinine ratio (ACR) was measured in mice placed in metabolic cages for the last 72 h of the study. Urine from the final 24 h-period was collected and analyzed for albumin and creatinine using the Albuwell M and Creatinine Companion assays (Exocell, Philadelphia, PA) [20]. To assess glomerular hypertrophy, perfused left kidney tissue was fixed in paraformaldehyde, paraffin-embedded, sectioned to 3 µm thickness, and stained with periodic acid-Schiff (PAS) stain [19]. Fifteen glomerular tufts per mouse were randomly chosen for hypertrophy analysis. Mesangial index was determined by calculating the PAS-positive area of each glomerulus relative to total area. MetaMorph was used for quantification of glomerular and PAS-positive areas and microscope images were captured using a digital camera, as per our published protocol [19].

### Statistical analyses

Statistical analyses were performed using GraphPad Prism 7 [21]. Normality of data was determined using Brown-Forsythe F-tests. Statistically significant differences (P < 0.05) for normally distributed data were analyzed using one-way ANOVA followed by Tukey’s post-test for multiple comparisons. Datasets were log2 transformed for non-normally distributed data, and the Brown-Forsythe F-test rerun. When log2-transformation normalized distribution, a one-way ANOVA followed by Tukey’s post-test for multiple comparisons was used. When log2-transformation did not normalize distribution, the non-parametric Kruskal-Wallis test, with Dunn’s post-test for multiple comparisons was used on the original, non-transformed dataset. Data are presented as mean ± standard error of the mean (SEM).

## Results

**Figure 2.**
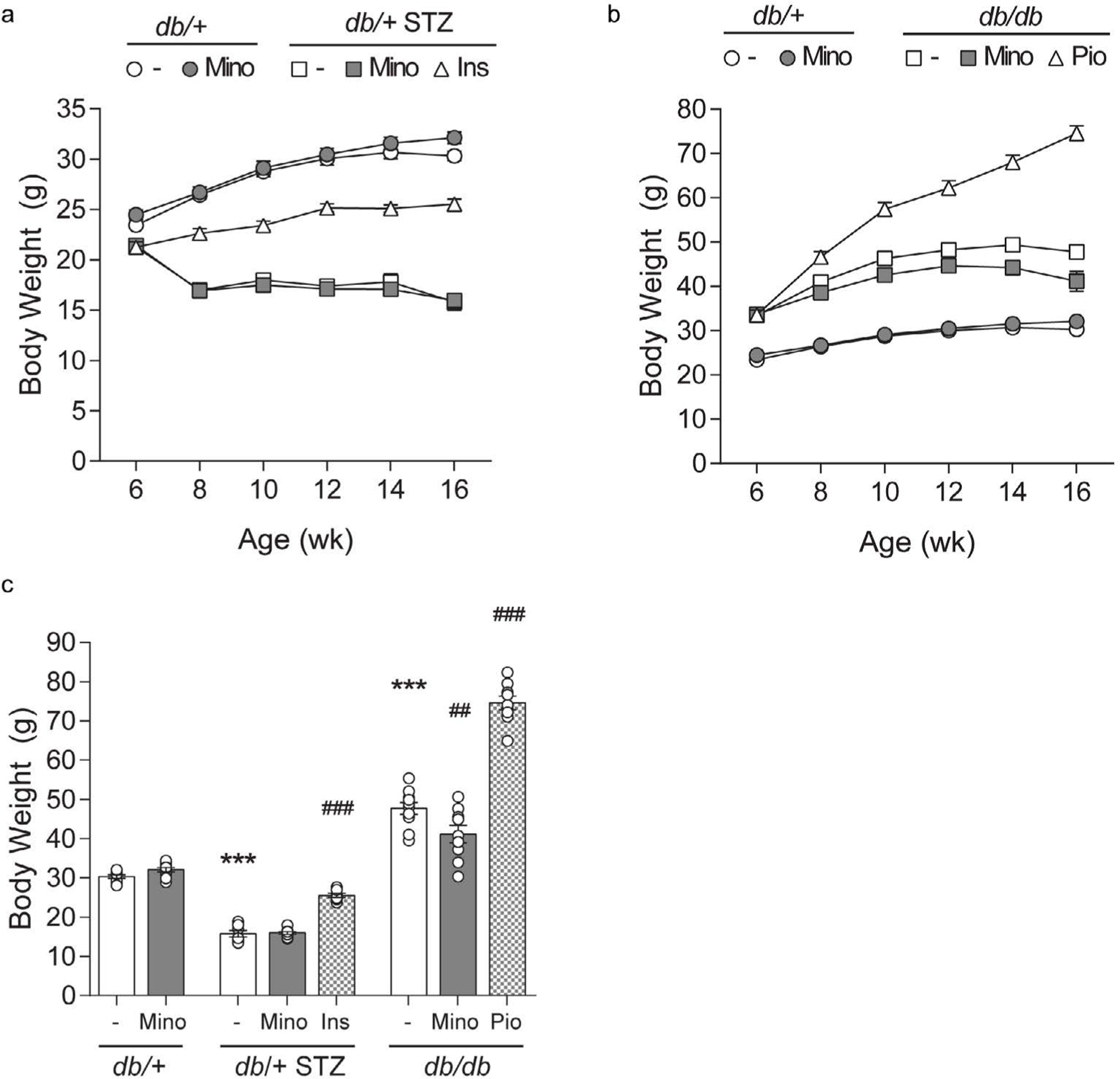
Longitudinal and terminal body weight. Body weights were measured at 6, 8, 10, 12, 14, and 16 weeks and are shown for control *db*/+ and *db*/+ MINO against: (A) T1D *db*/+ STZ, T1D *db*/+ STZ MINO, T1D *db*/+ STZ INS, and (B) T2D *db*/*db*, T2D *db*/*db* MINO, T2D *db*/*db* PIO. (C) Terminal body weights measured at 16 weeks. INS, insulin; MINO, minocycline; PIO, pioglitazone. Data are mean ± standard error of the mean (SEM). Two-way ANOVA with Tukey’s multiple comparisons test. ***P<0.001, versus *db*/+ controls; ##*P*<0.01, ###*P*<0.001, versus T1D *db*/+ STZ or versus T2D *db*/*db*; n = 7–10 mice

**Figure 3.**
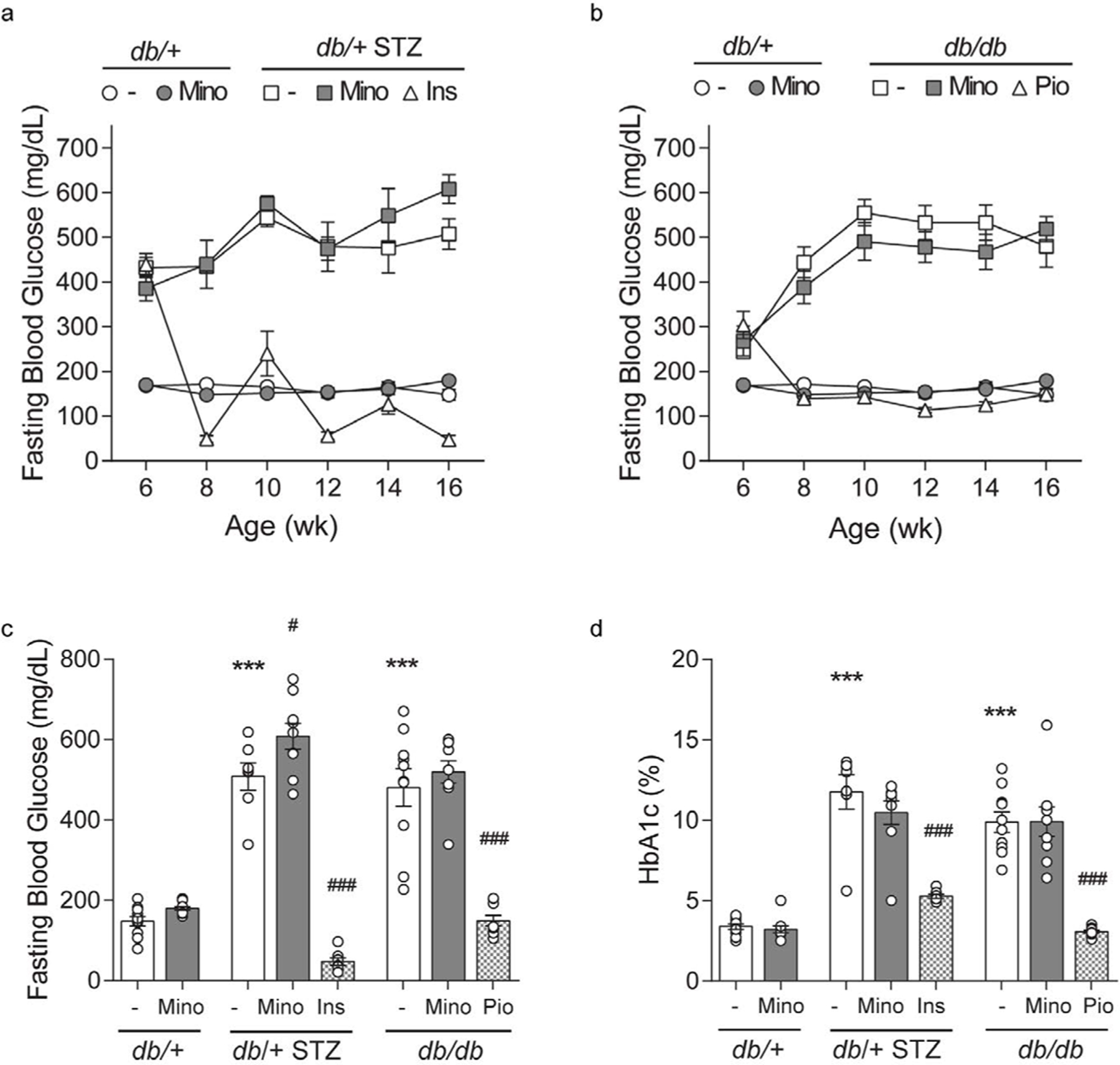
Longitudinal and terminal glycemic status. FBG was recorded at 6, 8, 10, 12, 14, and 16 weeks and is shown for control *db*/+ and *db*/+ MINO against: (A) T1D *db*/+ STZ, T1D *db*/+ STZ MINO, T1D *db*/+ STZ INSULIN, and (B) T2D *db*/*db*, T2D *db*/*db* MINO, T2D *db*/*db* PIO. Terminal (C) body weights and (D) percent glycated hemoglobin (%HbA1c) were measured at 16 weeks. INS, insulin; MINO, minocycline; PIO, pioglitazone. Data are mean ± SEM. Two-way ANOVA with Tukey’s multiple comparisons test. ***P<0.001, versus *db*/+ controls; #*P*<0.05, ###*P*<0.001, versus T1D *db*/+ STZ or versus T2D *db*/*db*; n = 7–10 mice

**Figure 4:**
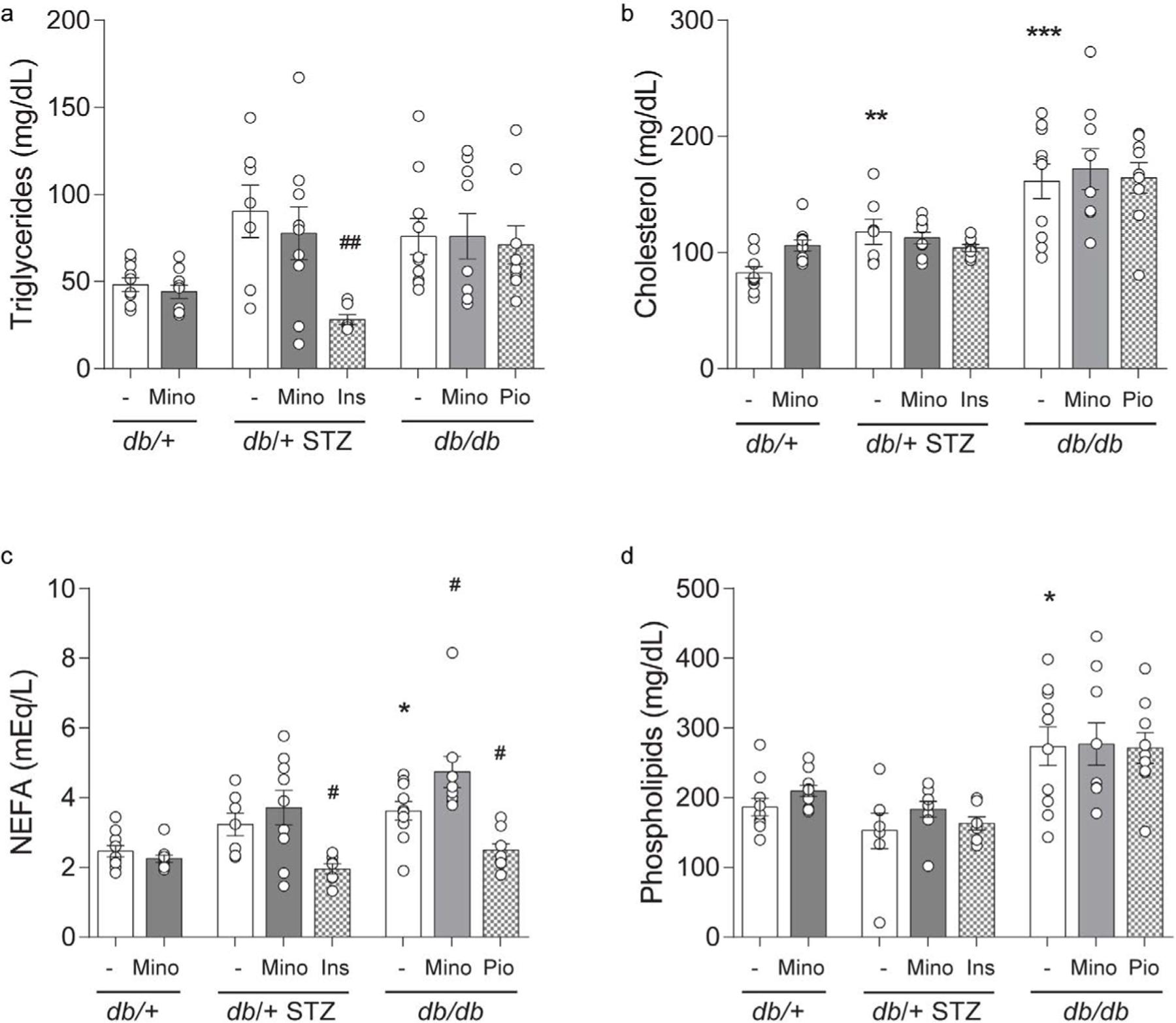
Terminal lipid profiles. (A) Triglycerides, (B) cholesterol, (C) non-esterified fatty acids (NEFAs), and (D) phospholipids were measured for all eight mouse cohorts at 16 weeks. INS, insulin; MINO, minocycline; PIO, pioglitazone. Data are mean ± SEM. Two-way ANOVA with Tukey’s multiple comparisons test. *P<0.05, **P<0.01, ***P<0.001, versus *db*/+ controls; #*P*<0.05, ##*P*<0.01, versus T1D *db*/+ STZ or versus T2D *db*/*db*; n = 7–10 mice

Minocycline only marginally affects metabolic phenotypes in T1D and T2D mice. Throughout the study, T1D db/+ STZ mice showed significant weight loss (Figures 2A and 2C) and elevated FBG (Figures 3A–3C) relative to nondiabetic controls (db/+). At study termination, the higher FBG was reflected by increased %HbA1c in T1D db/+ STZ mice versus controls (Figures 3C and 3D). The lipid profile of T1D db/+ STZ mice was broadly similar to db/+ controls, except they exhibited higher cholesterol levels, without differences in triglycerides, non-esterified fatty acids (NEFAs), and phospholipids (Figure 4). Minocycline did not affect body weight (Figure 2C), terminal %HbA1c (Figure 3D), or lipid profiles (Figure 4) in T1D db/+ STZ mice, although terminal FBG was marginally, though significantly, higher versus controls (Figure 3C). Minocycline also had no effect on control db/+ mice. In contrast, insulin partially restored terminal body weight (Figure 2C), FBG, and %HbA1c (Figures 3C and 3D) in T1D db/+ STZ mice relative to controls. It also significantly reduced triglycerides and NEFAs, but not cholesterol or phospholipids levels in T1D db/+ STZ mice versus controls (Figure 4).

During the experimental period, T2D db/db mice exhibited significant weight gain (Figures 2B and 2C) and raised FBG (Figures 3B and 3C) relative to control db/+ animals. At study conclusion, the higher FBG was paralleled by an elevated %HbA1c in T2D db/db mice versus controls (Figures 3C and 3D). T2D db/db mice had profound dyslipidemia, with increased cholesterol, NEFAs, and phospholipids, but unaltered triglycerides in comparison to controls (Figure 4). Minocycline slightly reduced terminal body weight (Figure 2C) but did not improve terminal glycemic control in T2D db/db mice (Figures 3C and 3D). It also slightly but not significantly perturbed NEFAs in T2D db/db mice (Figure 4C). Otherwise, minocycline was without effect on the metabolic profile in T2D db/db animals. On the other hand, pioglitazone exerted a greater influence on T2D db/db mice, markedly raising their weight (Figures 2B and 2C) but lowering their terminal FBG and %HbA1c to db/+ levels (Figures 3C and 3D). Pioglitazone also ameliorated NEFAs in T2D db/db mice but did not impact cholesterol or phospholipids (Figure 4). Overall, minocycline only marginally affected metabolic phenotypes in diabetic mice, whereas insulin and pioglitazone significantly improved multiple metabolic criteria in T1D and T2D mice, respectively.

### Minocycline does not improve DPN phenotypes in T1D and T2D mice

**Figure 5.**
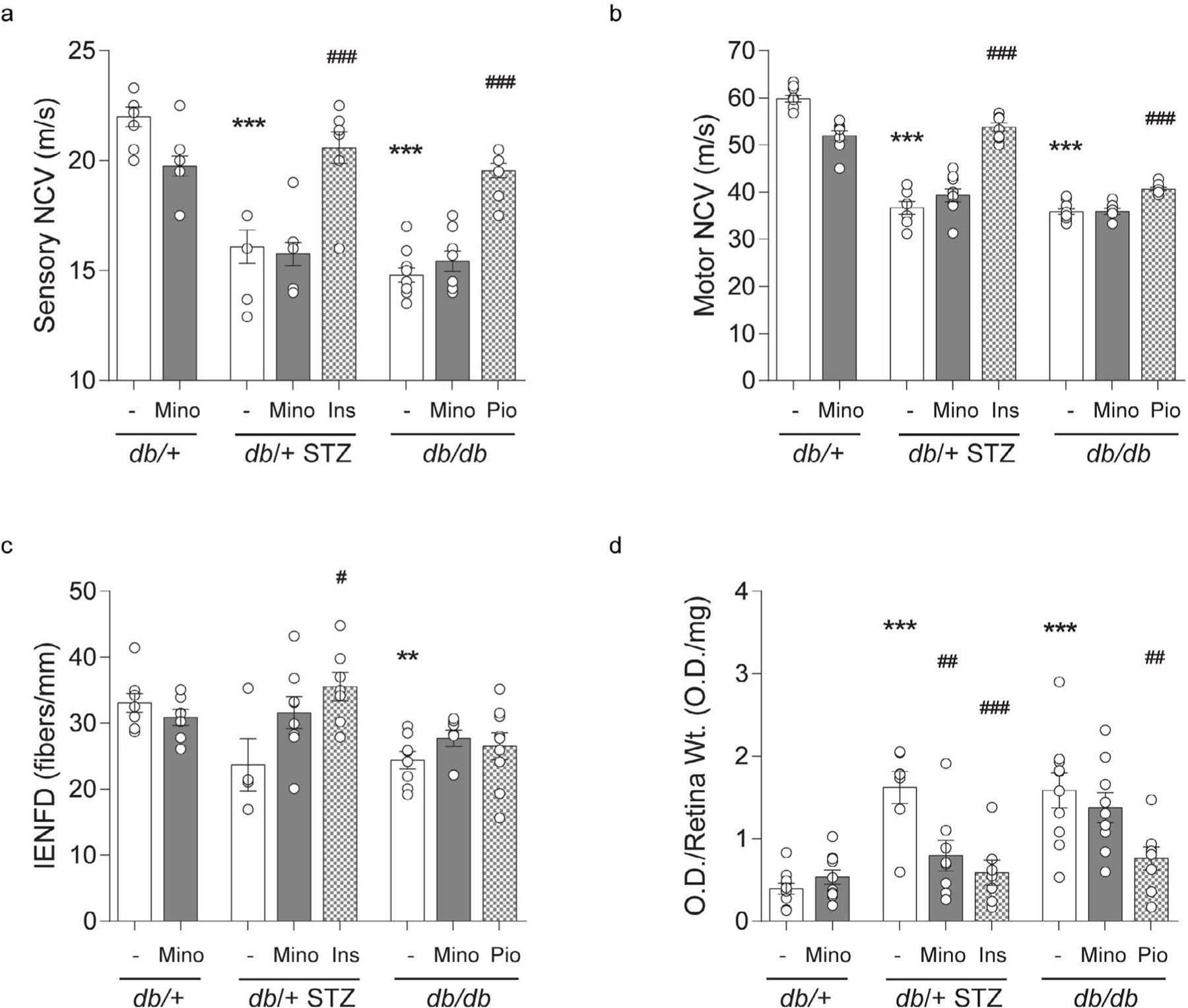
Terminal DPN and DR phenotyping. Terminal large-fiber nerve conduction velocities (NCVs) in (A) sural sensory and (B) sciatic motor nerves. (C) Terminal small-fiber intraepidermal nerve fiber density (IENFD). (D) Terminal apoptosis by retinal DNA fragmentation expressed as optical density from ELISA relative to wet retinal tissue weight. All metrics were measured for all eight mouse cohorts at 16 weeks. INS, insulin; MINO, minocycline; PIO, pioglitazone. Data are mean ± SEM. Two-way ANOVA with Tukey’s multiple comparisons test. **P<0.01, ***P<0.001, versus *db*/+ controls; #*P*<0.05, ##*P*<0.01, ###*P*<0.001, versus T1D *db*/+ STZ or versus T2D *db*/*db*; n = 4–10 mice

STZ-induced T1D db/+ mice display microvascular complications, including DPN, DR, and DKD [14,22,23]. As anticipated, T1D db/+ STZ mice had significantly decreased large-fiber sensory and motor NCVs relative to control db/+ animals, although their small-fiber IENFD only trended towards lower values (P = 0.052; Figures 5A–5C). Minocycline had no effect on any of these DPN metrics, in either T1D db/+ STZ or control db/+ mice. In contrast, insulin normalized large-fiber conduction and increased IENFD in T1D STZ db/+ mice relative to controls (Figures 5A–5C). We have previously reported that obese, leptin signaling-deficient T2D db/db mouse develops DPN [3,20]. As predicted, T2D db/db animals in this study displayed pronounced neuropathy in both large- and small-fiber measures versus controls (Figures 5A–5C). Minocycline did not affect the DPN phenotype in these T2D animals. However, pioglitazone did exert a significant effect on large-fiber NCVs. In summary, minocycline does not appear to improve DPN in diabetic mice, while insulin and pioglitazone significantly improved DPN phenotypes in T1D and T2D mice, respectively.

### Minocycline improves DR in T1D and DKD in T2D mice

Retinal apoptosis is a surrogate for DR progression, when quantified relative to wet retinal tissue weight. As anticipated, retinal apoptosis was increased in T1D db/+ STZ mice in relation to controls (Figure 5D). Minocycline lowered retinal apoptosis significantly in T1D db/+ STZ mice, as did insulin administration. Increased apoptosis was also observed in the retina of the T2D db/db mice (Figure 5D), which was ameliorated by pioglitazone but not by minocycline treatment. Thus, minocycline improved DR phenotype in the T1D but not the T2D mouse model.

**Figure 6.**
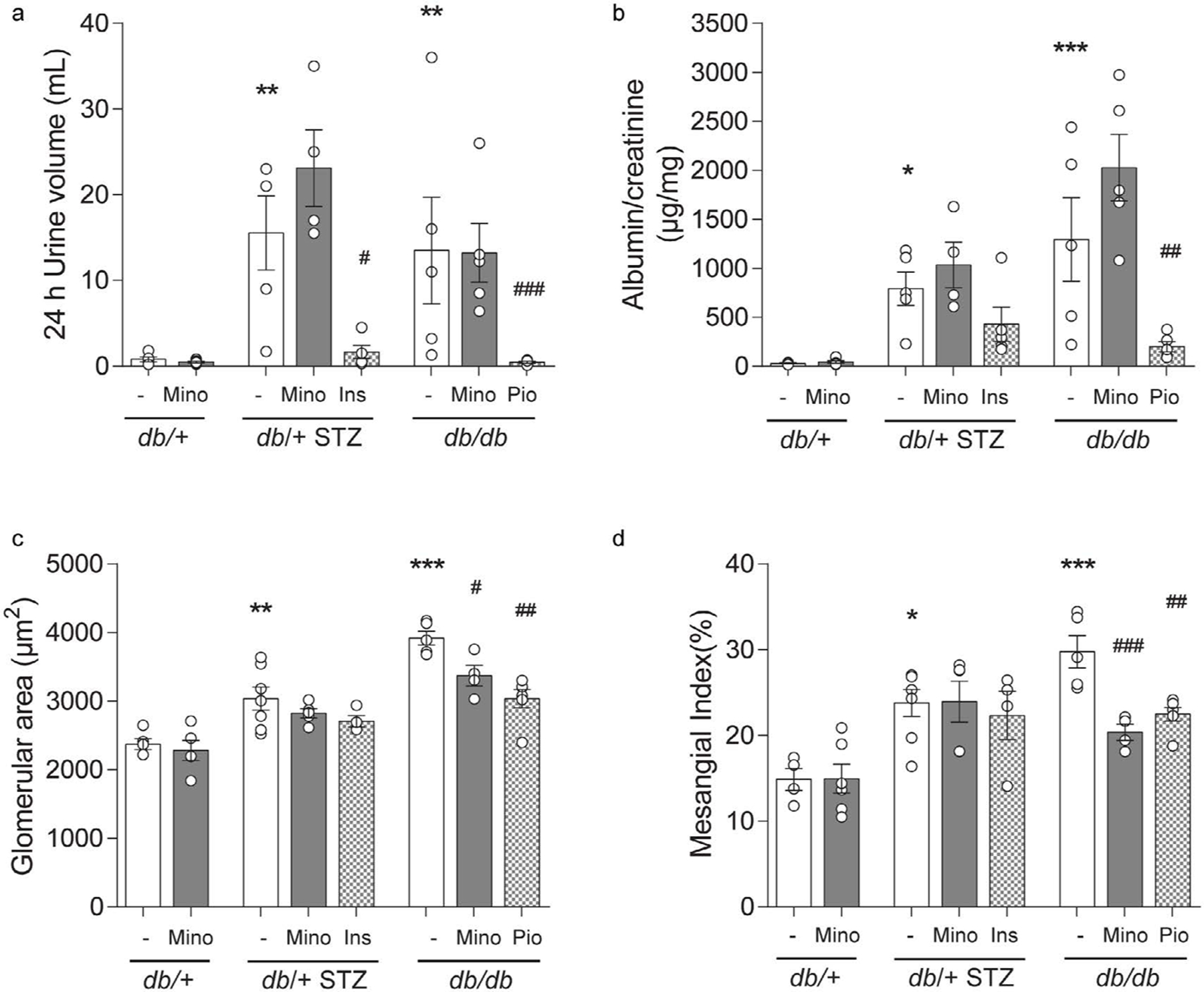
Terminal DKD phenotyping. Terminal metrics of renal function by (A) 24 h urine volume and (B) albumin-to-creatinine ratio. Terminal metrics of renal histopathology by (C) glomerular area and (D) mesangial index. All metrics were measured for all eight mouse cohorts at 16 weeks. INS, insulin; MINO, minocycline; PIO, pioglitazone. Data are mean ± SEM. Two-way ANOVA with Tukey’s multiple comparisons test. *P<0.05, **P<0.01, ***P<0.001, versus *db*/+ controls; #*P*<0.05, ##*P*<0.01, ###*P*<0.001, versus T1D *db*/+ STZ or versus T2D *db*/*db*; n = 4–7 mice

Both T1D db/+ STZ and T2D db/db mice exhibited elevated ACR compared to db/+ controls (Figures 6A and 6B), as well as increased glomerular area and mesangial index (Figures 6A and 6B). Minocycline had no effect on any of these parameters in T1D db/+ STZ mice but partially prevented glomerular hypertrophy and increased mesangial index in the T2D db/db animals. Minocycline did not affect ACR in the T2D db/db mice, while pioglitazone treatment prevented all aspects of DKD in the T2D model.

## Discussion

Multiple studies identify inflammation, oxidative stress, apoptosis, and dysregulated immunity and energy metabolism as consistent features in nerve, retinal, and kidney tissue in both mouse models and patients with T1D and T2D [3–6,24–32]. We and others [8–13,33–36] have consequently investigated minocycline, with its anti-inflammatory, anti-apoptotic, and anti-oxidant effects [7], as a possible therapeutic agent in DPN, DR, and DKD. Despite several reports showing that minocycline improved aspects of each individual complication [8,10–13,34–36], the current study represents the first time the simultaneous effects of minocycline on DPN, DR, and DKD were compared in a STZ-induced T1D model and a leptin signaling-deficient T2D model on a BKS background. We also contrasted the therapeutic efficacy of minocycline to insulin and pioglitazone in T1D db/+ STZ and T2D db/db mice, respectively.

By ten weeks into the study, T1D and T2D animals exhibited severe diabetes, including elevation in terminal FBG and %HbA1c (Figure 3), and perturbations in serum lipid profiles, particularly in the T2D animals (Figure 4). Minocycline did not affect body weight and FBG in our experimental T1D and T2D cohorts and had little effect on blood lipids which is consistent with previous reports in T1D rat [9,11,34,36,37] and T2D mouse [13] models and in humans [38]. Although we did not anticipate that minocycline would impact metabolic parameters, we predicted that it would prevent diabetic complications due to its anti-inflammatory, anti-apoptotic, and anti-oxidant effects [7]. Minocycline also possesses favorable pharmacokinetic (PK) properties, including good nervous system penetration and long serum half-life in humans [39]. However, the protective effects of minocycline on diabetic microvascular complications were modest, at best, and varied between diabetic models.

In our study, a 10-week regimen of minocycline (55 mg/kg in chow) had no effect on NCVs or IENFD, large- and small-fiber measures of DPN respectively in T1D db/+ STZ and T2D db/db animals (Figures 5A–5C). In agreement with our findings, 25 mg/kg minocycline daily for 8 weeks yielded no effects on NCV or IENFD deficits in STZ-induced T1D rats [37]. In contrast, minocycline improved sural NCVs and pain related behaviors at a dose of 40 mg/kg for 3 days [8]. Similar reports of minocycline efficacy on pain are reported after short treatment trials [9,33,40], albeit intrathecal minocycline (10 nmol) did not ameliorate mechanical allodynia, as assessed by Frey monofilament in 10-week-old mice [41]. While data are limited, a 6-week trial of minocycline (100 mg, twice daily versus placebo) in 50 T2D subjects with DPN resulted in improved vibratory sensation and decreased pain perception in both minocycline treated and placebo subjects, although the improvement was greater in the minocycline arm [38]. A caveat of this trial is that quantitative NCVs and IENFD were not assessed, a requirement for demonstrating drug efficacy in clinical trials of DPN according to the Toronto Expert Panel on DPN [42]. The variation in the mouse studies, and the results of this sole clinical trial, suggest a possible short-lived salutary effect of minocycline. Clearly, further studies in both mice and man are needed to definitively determine any long-term benefit of minocycline treatment. In contrast, insulin completely normalized small- and large-fiber DPN metrics in T1D db/+ STZ mice (Figures 5A–5C), as anticipated [2]. Surprisingly, however, pioglitazone improved large-fiber NCVs but not small-fiber IENFDs in T2D db/+ animals (Figures 5A–5C), an effect opposite to our previous observations in the same T2D model [26]. We anticipate this discrepancy between studies is related to a variation in gut microbiota that can affect the metabolic phenotype [43] and hence DPN, a possibility we are currently examining.

In the current study, minocycline decreased DNA fragmentation, a surrogate marker of DR, in the retina of T1D db/+ STZ but not of T2D db/db animals (Figure 5D). Our results agree with the positive effects of minocycline treatment on DR in a T1D STZ rat model dosed twice daily with minocycline (22.5 mg/kg) for 10 days [34], as well as T1D STZ rats treated with 2.5 or 5 mg/kg minocycline daily for 4 or 8 weeks [10,11], or 50 mg/kg daily for 4 weeks [35]. There was no effect of minocycline on T2D db/db mice (Figure 5D), and to our knowledge, there are no reports of minocycline treatment in T2D mouse models for comparison. We did observe that both insulin and pioglitazone lowered retinal apoptosis in T2D db/db group and T1D db/+ STZ animals, respectively, a result that was anticipated for the T1D model [44] (Figure 5D). We speculate the efficacy of minocycline in our T1D but not T2D cohorts is intrinsic to the type of diabetes. As with DPN, further studies are needed to confirm our single report on the lack of minocycline efficacy on DR in T2D and explore its differential effects in T1D and T2D.

In our study, the effect of minocycline on DKD progression was distinct in each model (Figure 6), with improvement in some aspects of DKD in T2D db/db mice, but without effect in T1D db/+ STZ animals. In agreement with our results, STZ-induced T1D rats receiving daily minocycline (50 mg/kg) for 4 weeks had no improvement in renal histological markers, as assessed by renal hypertrophy and tubulointerstitial fibrosis index [36]. In contrast, another study in T1D STZ rats that received 20 mg/kg minocycline daily for 4 or 16 weeks found a reduction in proteinuria [12], with lowering of renal apoptosis and glomerulosclerosis, although no change in glomerular area was noted. In contrast to our results in T1D, treatment of T2D db/db mice with minocycline partly prevented glomerular hypertrophy and mesangial expansion but did not affect ACR (Figures 6A and 6B). Our data agree with a parallel study on the T2D db/db model, where a 12-week regimen of daily minocycline (5 mg/kg) in mice aged 8 to 20 weeks improved renal histology (apoptosis by TUNEL, fractional mesangial area) [13]. Our data also agree with two human clinical trials, where minocycline treatment had no effect on ACR [38]. Thus, the clinical trials and our study suggest that minocycline has an effect on glomerular growth and mesangial matrix expansion but no effect on albuminuria. Specifically, minocycline may have a specific therapeutic effect in T2D on glomerular mesangial cells and/or glomerular macrophages that play a direct role in mesangial expansion, but little or no effect on podocyte alterations in early diabetes, changes that are generally manifested by albuminuria.

## Conclusion

In summary, the literature is inconsistent on the potential effects of minocycline on diabetic microvascular complications. Differences could arise from variation in rodent models (rat versus mouse), drug dose and therapy duration, disease duration and age of animals at treatment commencement, as well as type of diabetes. Our study determined the efficacy of minocycline in a side-by-side comparison in T1D and T2D mice on the same genetic background. We found that minocycline has some potential for ameliorating microvascular complications in T1D and T2D, with improvement in DR in the T1D mice and DKD in the T2D mice, with no discernible effect on DPN in either model. While glycemic control via insulin or pioglitazone in T1D and T2D cohorts, respectively, rescued microvascular complications to a greater extent than minocycline, there remains the possibility patients could receive potential benefit from adjunctive minocycline therapy, i.e., it is possible that minocycline could exert a greater effect over and above good glycemic/metabolic control. Minocycline was well-tolerated with few adverse events in both our study as well as the other reported rodent [8,10–13,34–36] and human studies [38,45–47], supporting its further study in well established, well phenotyped animal models of diabetes, as well as in man.

## Abbreviations:

DKD: Diabetic kidney disease
DPN: Diabetic peripheral neuropathy
DR: Diabetic retinopathy
STZ: Streptozotocin
T1D: Type 1 diabetes
T2D: Type 2 diabetes
